# Developing a wellbeing framework for employees working from home within a South African government entity

**DOI:** 10.3389/fpsyg.2026.1817111

**Published:** 2026-08-06

**Authors:** Refilwe Selesho, Mokgata Alleen Matjie

**Affiliations:** Department of Industrial and Organizational Psychology, University of South Africa, Pretoria, South Africa

**Keywords:** contextual psychology, employee wellbeing, flexibility, remote work, working from home, working from home wellbeing (WFHWB) framework

## Abstract

The COVID-19 pandemic fundamentally transformed work arrangements, accelerating the shift to remote work and reshaping employee wellbeing. While working from home (WFH) offered flexibility and autonomy, it also introduced challenges such as social isolation, anxiety, and reduced engagement. Guided by contextual psychology, which emphasizes the dynamic interaction between individuals and their environments, this study examines employee wellbeing in a public sector context and aims to develop a context-sensitive wellbeing framework. A qualitative, hermeneutic phenomenological design was employed within a constructivist–interpretivist paradigm. Semi-structured interviews were conducted with nine employees from a South African government entity who transitioned to WFH. Data were analyzed using Interpretative Phenomenological Analysis (IPA). Contextual psychology informed the interpretation by emphasizing how organizational, social, and personal conditions shape wellbeing. Trustworthiness was ensured through established qualitative rigor strategies. A central theme of “recommended approaches” emerged, comprising five subthemes: happiness, flexibility, clear employee engagement, training and development, and constant support. Findings indicate that wellbeing is context-dependent and influenced by organizational practices, technological readiness, and social connection. Hybrid work enhanced happiness through social interaction and positive emotions; flexibility promoted autonomy; engagement reduced role ambiguity; training improved competence; and support strengthened resilience. These dimensions informed the development of an integrated working from home wellbeing framework (WFHWB).

## Introduction

The COVID-19 pandemic of 2020 introduced profound and enduring challenges to employee health and wellbeing, specifically through work mode changes (Zwane and Oosthuizen, 2025). Beyond the direct health consequences and loss of life, the pandemic fundamentally reshaped how work is organized and experienced (Blank et al., 2023; Shuster and Lubben, 2022). One of the most significant shifts was the rapid transition from traditional office-based work to working from home arrangements, which altered employees' social, psychological, and professional environments (Marx et al., 2025). Various scholars found the transition to be positive for the relationship between working remotely and wellbeing (Dong et al., 2025; Hina et al., 2025). However, for many employees, this transition meant moving from socially interactive workplaces to isolated home-based work, often with limited preparation or support, thus affecting employee wellbeing (Dong et al., 2025; Gregersen et al., 2023; Xanthopoulou et al., 2007; Zhang and Deng, 2025).

Wellbeing is broadly understood as a positive evaluation and experience of one's life (Disabato et al., 2025; Tov, 2018), encompassing both feeling good and functioning effectively (Kong et al., 2024; Seligman, 2018). It is often used interchangeably with wellness and includes interrelated dimensions such as physical, mental, emotional, social, and spiritual wellbeing (Schramme, 2023; World Health Organization, 2018). Within organizational contexts, employee wellbeing refers to a holistic experience of health, happiness, purpose, and effective stress management, contributing to both personal flourishing and enhanced organizational performance (Zwane and Oosthuizen, 2025). The literature distinguishes between two dominant approaches: hedonic wellbeing, which reflects subjective life satisfaction and affective states, and eudaimonic wellbeing, which emphasizes personal growth, meaning, and psychological functioning (Baselmans and Bartels, 2018; Martela and Ryan, 2023; Ryff, 1989; Van Dierendonck and Lam, 2023). Both dimensions are essential for understanding workplace wellbeing, particularly in times of crisis. During the COVID-19 pandemic, employee wellbeing was significantly influenced by work–family conflict, job stress, adaptability challenges, and organizational responses (Schifano et al., 2023; Vyas-Doorgapersad, 2023). However, it should also be noted that external, organizational, and personal factors may also determine the experience of wellbeing while working from home and the severity of the experiences (Blank et al., 2023). Hence, the current study opted to understand all three factors that might affect the wellbeing experiences of these employees at the entity. Studies have found that humans are social beings, and isolation may have negative psychological or mental health ramifications (Dong et al., 2025), including stress, anxiety, and depression (Peçanha et al., 2025; Schifano et al., 2023). Schifano et al. (2023) concluded that employees working from home during COVID-19 had anxiety and thus experienced low wellbeing levels. Moreover, such negative experiences can negatively affect organizational outcomes, including reduced engagement, productivity, and increased turnover intentions (Nair et al., 2025).

Despite these risks, there is a dearth of a framework to assist employees working from home in the event of a COVID-19-like incident in the future. A framework will be used as a guide to employers when implementing working-from-home policies in the future. Collectively, the literature positions employee wellbeing as a dynamic, multidimensional construct shaped by both internal psychological processes and external organizational conditions (Blank et al., 2023). The pandemic underscored the interdependence of these dimensions, revealing how financial insecurity, social isolation, environmental changes, and psychological strain interact to influence overall flourishing (Helliwell et al., 2021; Hina et al., 2025). A comprehensive wellbeing framework must therefore integrate hedonic and eudaimonic components while addressing contextual and structural influences that shape employee experiences. Moreover, working from home mode, as enhanced by the technological advances, is here to stay (Battisti et al., 2022; Dong et al., 2025; Munyeka, 2024). As a result, it was deemed necessary, based on employees' experiences and challenges, to develop a comprehensive wellbeing framework for the South African context, especially the public entities sector. By situating employee wellbeing within its organizational and social context (Blank et al., 2023), the study seeks to deepen understanding of how working-from-home arrangements influence wellbeing in a crisis-driven work environment.

Working remotely is becoming a trend and is expected to endure with home office becoming integral to the work setting, and requires employers to understand employees' preferences on their preferred work locations (Gangisetty et al., 2024). Employees feel gratitude toward employers who allow them to work remotely and perceive the employer as caring and thoughtful, remain motivated, and perform optimally (Iqbal and Barykin, 2021; Maluleke, 2024). Some drawbacks of working from home, such as missed deadlines and employees being unavailable during critical times, were identified, which made it difficult for employers to keep track of employees' availability and performance (Gangisetty et al., 2024). Working from home in developing countries like South Africa was triggered by the COVID-19 pandemic (Schifano et al., 2023). Working from home now represents a core modality across sectors worldwide (Ketter et al., 2025), with little knowledge on how working from home and employee wellbeing intersect.

Within psychology, wellbeing is widely understood as encompassing subjective experiences of life satisfaction and positive affect, alongside psychological functioning such as resilience and engagement (Diener, 1984; Ryan and Deci, 2001; Ryan et al., 2022). In organizational contexts, wellbeing contributes not only to healthy psychological states but also to job performance, organizational commitment, and sustainable participation in work roles (Bakker and Demerouti, 2017; Bakker et al., 2023). The structural shift toward working from home demands renewed theoretical clarity on how wellbeing emerges in digital and blended work environments. Psychologically, the study will employ various theories to help explain wellbeing and will be used to develop the theoretical framework. This study adopts an integrated theoretical approach to develop a working-from-home wellbeing framework by drawing on multiple complementary perspectives. Contextual psychology provides the overarching lens, enabling the interpretation of employee wellbeing as embedded within organizational and social environments, particularly in crisis-driven remote work contexts. Building on this, multidimensional wellbeing is conceptualized through Ryff's Psychological WellBeing Model, which emphasizes optimal functioning (e.g., autonomy, relationships, and personal growth) (Ryff, 1989; Ryff and Keyes, 1995; Van Dierendonck and Lam, 2023), and the PERMA Model, which captures flourishing through positive emotion, engagement, relationships, meaning, and accomplishment (Al-Hendawi et al., 2024; Kovich et al., 2023; Seligman, 2011). From a work-specific perspective, Warr's Model of Mental Health highlights the role of job characteristics such as autonomy and competence in shaping employee mental health (Li et al., 2024; Warr, 1987, 1994). Additionally, Maslow's Hierarchy of Needs provides a motivational foundation by linking employees' wellbeing to the fulfillment of hierarchical needs, from basic security to self-actualisation (Dong et al., 2025; Maslow, 1943; McLeod, 2024). Together, these theories enable a holistic understanding of wellbeing that incorporates psychological functioning, workplace conditions, motivation, and lived experience, thereby informing the development of a contextually grounded and multidimensional working-from-home wellbeing framework.

The study was conducted at a public entity in South Africa, meaning the entity is state-owned and operates more or less like most government departments, with little technological advancement or transformation (Mofokeng et al., 2025). Research on public sector transformation notes that government organizations often lag behind the private sector in digital readiness and capacity for rapid change, resulting in slower uptake of digital tools and working from home technologies (Shibambu, 2024). As a result, the entity was most hit by the sudden working remotely situation and had to procure laptops (computers) and software licenses to enhance working from home. This abrupt shift aligns with broader evidence showing that when organizations rapidly adopt digital tools without prior planning or adequate support, resistance and integration challenges emerge because the change is unexpected and resources are scarce (Shibambu, 2024). This might mean that resistance was encountered due to unknown and unplanned change, scarce resources, and lack of training (Rafiq et al., 2022; Waqanimaravu and Arasanmi, 2020; Yertas, 2024), and, most importantly, fear of working alone, in isolation, which impairs employees' wellbeing negatively (Dong et al., 2025). As a result, the current study aimed to:

*Based on participants' wellbeing experiences and challenges encountered, develop a theoretical framework for the employees working at the South African entity to ensure their wellbeing*.

## Methods

This study adopted a qualitative research design underpinned by a hermeneutic phenomenological approach to explore and interpret the lived experiences of employees transitioning to working from home (van Manen, 2017; van Mannen, 2023). This implies that other than the wellbeing experiences and challenges experienced by employees when working, the researchers focused on the iterative “recommended solutions” for developing the theoretical wellbeing framework, as the “reality exists from the participants' lived experiences.” This design was appropriate for gaining an in-depth understanding of the subjective meanings participants attach to their wellbeing experiences. The study was grounded in the constructivist–interpretivist paradigm, which assumes that reality is socially constructed and context-dependent (Creswell and Creswell, 2023). Within this paradigm, constructivism recognizes that individuals develop personal meanings through interaction with their environments, while interpretivism emphasizes understanding these meanings from participants' perspectives (Creswell and Creswell, 2023). This lens allowed the researchers to capture the complexity and nuance of employees' emotional and psychological wellbeing within their social and organizational contexts.

### Participants and setting

Participants were selected through non-probability convenience sampling, focusing on employees from a government entity in Gauteng Province, South Africa, who had transitioned from office-based to working from home (Mutki, 2025; Stratton, 2023). Recruitment continued until data saturation was reached at the ninth participant. The eligibility criteria required participants to have experienced working from home to ensure they could provide meaningful reflections on their emotional and psychological wellbeing. The sample comprised diverse staff members across functional areas, offering a balanced representation of experiences within the organizational context.

### Data collection

Data were collected through semi-structured interviews to gain insight into the psychological and emotional challenges associated with working from home transitions. Each interview lasted approximately 45 min, was conducted in English, audio-recorded with consent, and transcribed verbatim. To ensure trustworthiness and rigor, the study adhered to established qualitative research criteria, including credibility, dependability, confirmability, and transferability (Creswell and Creswell, 2023; Kakar et al., 2023; Lincoln and Guba, 1985). Credibility was strengthened through detailed field notes, member checking, and reflexivity (Lloyd et al., 2024). Dependability was enhanced by prolonged engagement with the data and comparison with existing literature (Denzin and Lincoln, 2018; Kakar et al., 2023). Confirmability was ensured through peer review by the second author, while transferability was supported by providing comprehensive methodological details for replication (Kim, 2025). Participants provided informed consent and were assured of confidentiality and voluntary participation throughout the research process.

### Procedure

Ethical clearance was granted by the University of South Africa's College of Economics and Management Sciences ERC, Industrial and Organizational Psychology (Ref: 1428). Permission to access participants was obtained from the management of the participating government entity. All interviews were conducted by an experienced qualitative researcher skilled in phenomenological interviewing techniques and probing, ensuring depth and richness of data while maintaining participants' psychological safety.

### Data analysis

Data were analyzed using Interpretative Phenomenological Analysis (IPA), which integrates phenomenology, the study of lived experience, with hermeneutics, the theory of interpretation (van Manen, 2017; van Mannen, 2023; Willis and Harvey, 2025). This approach enabled the researchers to examine both individual meanings and shared experiential patterns. The analysis followed an iterative process of reading, coding, and thematic abstraction. Themes were named to accurately capture their essence and connection to participants' narratives (Polat, 2025). To enhance dependability, the researchers provided detailed descriptions of analytic procedures and maintained an audit trail (Kakar et al., 2023). The second author independently reviewed and validated the emergent themes to ensure analytical rigor and interpretive coherence.

## Results and discussion

The manuscript was adapted from a bigger study, which had other themes such as *wellbeing experiences* and *wellbeing challenges*, which led to the current theme of *recommended approaches for working from home wellbeing*. The study aimed to develop a theoretical framework based on which organizations can manage working from home while ensuring comprehensive employee wellbeing. Over and above the experiences, challenges, and effects, we went further and asked the participants which approaches they think will work to promote employees' wellbeing while working remotely. As a result, the following theme, “*recommended approaches”* and subthemes, namely, *happiness, flexibility, clear employee engagement, constant support, training, and development* emerged (see Table 1 below), and we used these subthemes to develop the theoretical framework.

**Table 1:** Research question, themes, and subthemes.

Research question	Theme	Subthemes
Which approaches do they think will work to promote employees' wellbeing while working remotely?	Recommended approaches	1 Happiness 2 Flexibility 3 Clear employee engagement 4 Constant support 5 Continuous Training and development

### Subtheme 1: happiness

When asked to recommend strategies that work to enhance their wellbeing as employees working from home, participants shared that the introduction of a hybrid working mode might lead to happiness by reducing stress and anxiety, and help employees cope with total working from home. Participant 1 shared that: “*I wouldn't really mind if we could have a hybrid model where there was more flexibility now because we work a lot from home so I really would prefer if we had a hybrid model.”* And this was supported by P5 by adding that they need to interact with colleagues: “*I think hybrid is a good way because you know, to work totally from home, we don't want that. I mean, he wants to be able to interact with colleagues. So, I don't think I wouldn't say work from home. It's good to be in the office.”* It is thus evident that one of the solutions to total or permanent working from home is a hybrid work mode because it leads to happiness, which is related to PERMA's positive emotions and Maslow's higher-order needs of esteem and self-actualization (Maslow, 1943; McLeod, 2024; Seligman, 2018). Employees in hybrid working arrangements tend to become happy employees or experience more frequent positive emotions because they get to interact with their colleagues at least a few days a week, as compared to full working from home mode (Dong et al., 2025; Maslow, 1943).

### Subtheme 2: flexibility

Although mentioned by two participants, this subtheme emerged and showed novelty because most organizations focused and invested in information and technology infrastructure during the COVID-19 pandemic, neglecting the other crucial parts of the organizations, such as the human resources. P2 indicated that for the overall wellbeing of the organizations, it has to be fully flexible by adapting to new technology in totality, as shown here:

“*I would think um maybe have a sort of flexi-time working arrangements or a hybrid working model if that is what you can call it so I remember back then just after the lockdown we had sort of a rotation if you can put it like that.” (P3)*

and

“*A hybrid working model. Maybe 3 days office, 2 days in, and then we alternate among the colleagues within the unit. That's also, and whoever wants to be in the full office, it's fine, let them be, but just have three in the office and 2 days working from home.” (P2)*

These findings are supported by previous literature that concluded that employees' flexibility enhances their psychological wellbeing, among others (Petcu et al., 2023; Ryan and Deci, 2020; Ryan et al., 2022). Flexible employees experience less anxiety according to Schifano et al. (2023) because they become autonomous and can fulfill their aspirations fully (Li et al., 2024; Warr, 1987, 1994). Moreover, such employees can reach their highest order needs according to Maslow (1943) and Arief and Amrullah (2025), as well as Dong et al. (2025).

### Subtheme 3: clear employee engagement

Another novelty that is often taken for granted in most corporate organizations is the need for clear employee engagement. This is necessary to avoid misinformation and poor performance and promote employee engagement. The entity should, according to the participants, ensure clear employee engagement between management and employees, as shared here:

**“** *And they must also, I feel like they also need to engage with all the employees, for example, in terms of management's decision to return to the office arrangements. Employees are motivated when feel engaged.” (P2)*

Clear and regular engagement makes employees feel connected to the organization, which in turn enhances emotional connections leading to wellbeing as per PERMA's engagement relationship dimensions (Seligman, 2011). Moreover, clear constant engagement ensures that employees understand their role clearly as queries are addressed immediately, minimizing role ambiguity (Li et al., 2024; Warr, 1987).

### Subtheme 4: training and development

Six participants indicated that they require training and development with the new and latest technologies they are using while working remotely.

“*It was an unknown territory, not knowing how to work from home or how to do it when you haven't done it before, especially working online and online meetings. I And in my particular instance, the type of work that I do, how do I do it from home”? (P4)*

“*Yes, it was challenging, especially with the technologies that we had to adjust to. We had to adjust. Yes. Using the laptop and the MS Teams for meetings.” (P6)*

*During the start of the COVID-19 pandemic, communication tools such as Zoom and MS Teams were introduced and some of us didn't know how to make use of them…..anyway, training was provided but it would have been helpful if the organization trained the employees on how to work on these platforms…..I guess no one was aware that the pandemic will hit us…but I must say we have learnt a lesson. It is important for the organization to implement continuous training and development programmes on new technologies.” (P5)*

The training will not only lead to their productivity but also to personal development (Ryff and Keyes, 1995; Van Dierendonck and Lam, 2023), personal growth and mastery, and competence (Li et al., 2024; Warr, 1978) to contribute meaningfully (Seligman, 2011). Studies showed that employees display a higher level of motivation and wellbeing when they are constantly trained and developed (Li et al., 2024; McAnally and Hagger, 2024; Ryff and Keyes, 1995; Warr, 1987; Van Dierendonck and Lam, 2023). Training and development are defined as processes that provide employees with methodical development of abilities, knowledge, and attitudes required to perform successfully in their job to achieve organizational goals (Oyenuga et al., 2024). According to Waqanimaravu and Arasanmi (2020), continual employee training and development programmes are one of the major methods of upgrading and enhancing employees' skills, proficiencies, and understanding in the workplace, as supported recently by Rafiq et al. (2022) and Yertas (2024).

### Subtheme 5: constant support

Five participants mentioned the importance of constant support during the crisis, although only one pertinent quote is quoted under this subtheme:

**“** *It wouldn't be just like oh, so and so has COVID-19 and you move on with your life. Indirectly or indirectly it has affected me. It affected me yeah….and not speaking for myself only, most of our colleagues, including me, needed support from either our immediate managers or the organization itself.” (P7)*

The PERMA model supports this theme through its relationships dimension, which supports the need for employees to engage and support each other through their relationships for a sense of belonging and safety (Dong et al., 2025; Maslow, 1943). Engagement refers to being interested, attracted, and involved in an activity, whereas relationships are about feeling supported, valued, and loved by others (Al-Hendawi et al., 2024; Seligman, 2011). Researchers emphasized the need for constant support in the workplace because it enables social and psychological functioning or affective wellbeing (Li et al., 2024; Warr, 1987, 1994) and mitigates stress reactions (Hina et al., 2025; Jolly et al., 2020), and they also facilitate employees' skills to support each other to decrease the perceived work-related stress (Bakker et al., 2023; Hina et al., 2025). Supportive employees motivate and enhance one another's wellbeing, reduce burnout in the workplace, and contribute to the achievement of basic needs such as autonomy, relatedness, and competence (Bakker et al., 2023; Hina et al., 2025; Mathieu et al., 2019).

### The developed wellbeing framework

The main aim of this study was to develop a working-from-home wellbeing framework. Various theories were used as foundational models, and participants' responses were linked to various dimensions of the theories to create the framework, as seen below.

**Figure 1:**
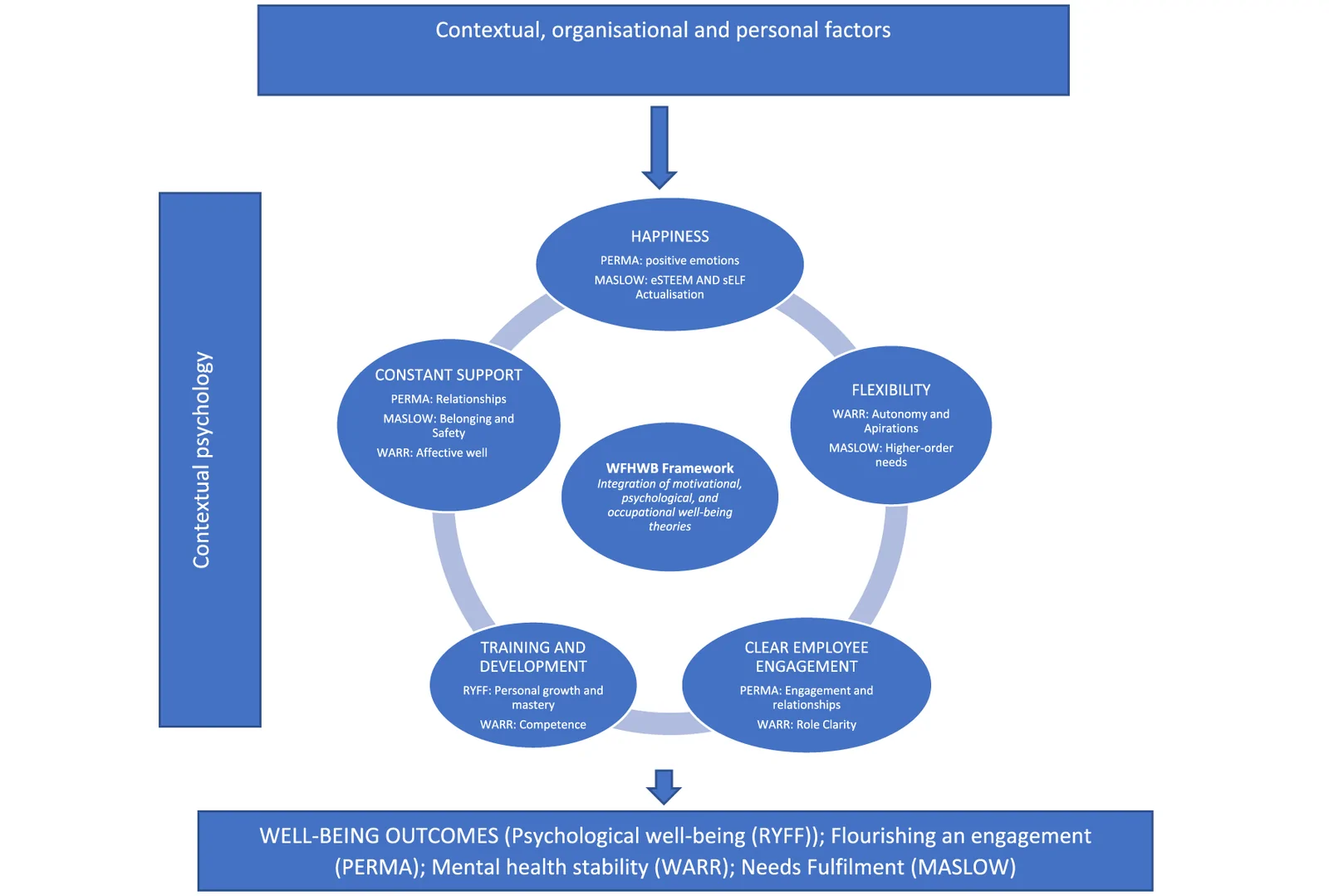
Working from home wellbeing (WFHWB) framework.

As noted in Figure 1 above, employee wellbeing has emerged as a critical area of focus in contemporary organizational research, particularly with the rise of remote and hybrid work structures. Increasingly, scholars argue that wellbeing is not a singular construct but a multidimensional phenomenon encompassing emotional, psychological, and occupational dimensions. This essay critically examines an integrated WFHWB framework that draws on four major theoretical perspectives: Maslow's (1943) hierarchy of needs, Ryff's (1989) psychological wellbeing model, Warr (1987)'s occupational wellbeing framework, and Seligman's (2011) PERMA model. The framework conceptualizes wellbeing across five key dimensions: happiness, flexibility, employee engagement, training and development, and constant support.

At its core, the framework recognizes that *happiness* represents both a hedonic and eudaimonic experience. Within Seligman's (2011) PERMA model, positive emotions constitute a central pillar of wellbeing, encompassing feelings such as joy, contentment, and optimism (Kovich et al., 2023; Seligman, 2011). These emotional states are not merely outcomes but actively contribute to resilience and improved cognitive functioning. However, the framework extends beyond hedonic wellbeing by incorporating Maslow's (1943) higher-order needs of esteem and self-actualisation (ultimate happiness), a notion supported by a recent study by Arief and Amrullah (2025). From this perspective, happiness is not limited to transient emotional states but includes a deeper sense of fulfillment derived from achievement and personal growth (Arief and Amrullah, 2025; Maslow, 1943). By integrating Maslow's (1943) theory, the framework addresses this limitation, linking emotional wellbeing with broader human developmental needs, supported by Arief and Amrullah (2025).

The dimension of *flexibility* reflects the growing importance of autonomy in remote work environments. Warr's (1987) model highlights autonomy as a key determinant of occupational mental health, suggesting that employees who have control over their work conditions experience higher levels of satisfaction and lower levels of stress (Li et al., 2024). Similarly, Maslow's (1943) theory's conceptualization of higher-order needs emphasizes independence and self-direction as essential for self-actualisation (Arief and Amrullah, 2025). Contemporary research supports this view, indicating that flexible work arrangements enhance intrinsic motivation and overall wellbeing (Yildizhan et al., 2023). However, flexibility is not universally beneficial; excessive autonomy without adequate structure can lead to role ambiguity and work intensification (Vermeer, 2025). Thus, while the framework rightly positions flexibility as a core dimension, its effectiveness is contingent upon employee engagement and organizational support systems.

*Employee engagement*, as conceptualized in the framework, integrates both psychological immersion and structural clarity. Within PERMA, engagement refers to deep involvement or “flow” in work activities, which is associated with increased productivity and satisfaction (Kovich et al., 2023; Seligman, 2011). At the same time, Warr's (1987) emphasis on role clarity underscores the importance of clearly defined expectations in reducing stress and enhancing performance (Li et al., 2024). This dual perspective is particularly relevant in remote work contexts, where physical separation can exacerbate uncertainty. Nevertheless, a critical limitation lies in the assumption that engagement is always beneficial. Emerging research suggests that excessive engagement may lead to burnout, particularly in digitally intensive work environments (Gregersen et al., 2023; Xanthopoulou et al., 2007; Zhang and Deng, 2025). Therefore, engagement should be balanced with recovery and boundary management.

The framework's inclusion of *training and development* highlights the importance of continuous learning for sustainable wellbeing. Ryff's (1989) model conceptualizes wellbeing in terms of personal growth and environmental mastery, emphasizing the individual's capacity to develop skills and adapt to challenges, as supported by Van Dierendonck and Lam (2023). Warr (1987) and Li et al. (2024) complement this perspective by identifying competence as a key factor in occupational wellbeing. Together, these theories suggest that employees who perceive growth opportunities are more likely to experience a sense of purpose and satisfaction. However, a critical consideration is that access to training is often unevenly distributed across organizations, potentially reinforcing inequalities. As such, the effectiveness of this dimension depends on equitable and inclusive organizational practices (Rafiq et al., 2022; Waqanimaravu and Arasanmi, 2020; Yertas, 2024).

Finally, *constant support* is presented as a foundational dimension that underpins all other aspects of wellbeing. Seligman's (2011) PERMA model identifies relationships as essential to flourishing, a finding supported by Kovich et al. (2023), while Maslow highlights belongingness and safety as fundamental human needs, a finding supported by Arief and Amrullah (2025). Warr (1987)'s concept of affective wellbeing further emphasizes the role of supportive environments in shaping emotional experiences at work, supported by Li et al. (2024). In remote contexts, where social isolation is a significant risk, organizational support becomes even more critical (Al-Hendawi et al., 2024; Bakker et al., 2023; Dong et al., 2025; Hina et al., 2025; Jolly et al., 2020). However, the framework could be strengthened by distinguishing between different types of support (e.g., managerial, peer, and organizational) and recognizing that support must be perceived as genuine to be effective.

Overall, the WFHWB framework offers a comprehensive and integrative approach to understanding employee wellbeing. Its primary strength lies in combining multiple theoretical traditions to capture the complexity of wellbeing in modern work environments. By linking emotional experiences (PERMA), developmental processes (Maslow and Ryff), and work design factors (Warr), the framework provides a holistic understanding that aligns with contemporary research. However, its application requires careful consideration of contextual factors such as organizational culture, leadership practices, and technological demands.

In conclusion, employee wellbeing in remote work settings is shaped by a dynamic interplay of psychological, social, and organizational factors. The integrated framework discussed in this essay effectively demonstrates that wellbeing is not merely the absence of distress but the presence of positive functioning, growth, and support.

### Limitations

While this study provides valuable insights into the lived experiences and wellbeing of employees adapting to working from home, several limitations should be acknowledged. The use of a qualitative hermeneutic phenomenological design, although wellsuited to exploring participants' subjective experiences, along with the non-probability purpose and convenience sampling of a small sample size (*N* = 9), all drawn from a single government entity in Gauteng Province. This makes the findings context-specific, thus not generalizable to other contexts. However, it should be noted that the qualitative studies focus on depth rather than generalization, as recently noted and supported by seminal and recent researchers (Guba, 1981; Guba and Lincoln, 1989; Megheirkouni and Moir, 2023; Gamble and Hewlett, 2025). The constructivist-interpretivist paradigm emphasizes the co-construction of meaning between researcher and participant, which inherently introduces a degree of researcher subjectivity. However, according to McKim (2023), reflexivity and member-checking are trusted, useful tool enhance credibility as they were employed in this study. While semi-structured interviews allowed rich, in-depth narratives, the reliance on self-reported data may have introduced recall bias or social desirability effects, particularly when discussing personal wellbeing challenges. However, the researchers were careful to self-bracket and base all their findings on existing literature rather than personal experiences, as confirmed by Saime et al. (2025).

### Implications

#### Theorical

The proposed framework extends theoretical understandings of working from home wellbeing by integrating motivational, humanistic, and positive psychology perspectives. From a theoretical standpoint, the synthesis of the theories provided a multidimensional understanding of how individual motivation, basic psychological needs, and flourishing interact in virtual work settings. This integration advances organizational psychology by positioning wellbeing not as an individual responsibility, but as a systemic outcome supported by structural autonomy, relational connectedness, and continuous competence development (Arief and Amrullah, 2025; Dolan et al., 2021; Lin et al., 2024; Maslow, 1943; Ryff, 1989; Seligman, 2011; Warr, 1987). The model further contributes to theory by highlighting the reciprocal nature of wellbeing components, where engagement and support enhance happiness, and in turn, happiness reinforces engagement and performance.

#### Empirical

This framework provides an empirically informed foundation for future research on working from home wellbeing by bridging psychological theory with observable organizational practices. Empirically, it contributes to the growing body of evidence linking working from home design to employee motivation, satisfaction, and performance outcomes. Studies have shown that autonomy, supportive engagement, and professional development are strong predictors of remote employees' engagement and wellbeing (Al-Hendawi et al., 2024; Bakker et al., 2023; Dong et al., 2025).

#### Practical

Practically, the framework provides actionable guidance for organizations seeking to foster sustainable working from home environments. Employers should prioritize clear, constant engagement structures, consistent emotional and technical support, and ongoing digital skills training to maintain competence and connection (Li et al., 2024; McAnally and Hagger, 2024; Oyenuga et al., 2024; Rafiq et al., 2022; Van Dierendonck and Lam, 2023; Yertas, 2024). Policies that promote flexibility and autonomy, such as hybrid scheduling and outcome-based performance measures, can enhance intrinsic motivation and work-life integration as suggested and supported by (Arief and Amrullah, 2025; Li et al., 2024; Vermeer, 2025; Yildizhan et al., 2023). Leadership development initiatives should also emphasize empathy, inclusion, and psychological safety to strengthen belonging and trust. Ultimately, by operationalising these principles, organizations can cultivate a working-from-home culture grounded in wellbeing, engagement, and productivity, aligning human flourishing with organizational resilience and long-term success (Al-Hendawi et al., 2024; Bakker et al., 2023; Dong et al., 2025; Hina et al., 2025; Jolly et al., 2020; Kovich et al., 2023).

## Conclusions and recommendations

This study *aimed to explore employee's wellbeing experiences and challenges while working from home and based on employees' recommended solutions or approaches, then develop a theoretical WFHWB framework*. The development of a theoretical framework was based on the approaches suggested by the participants, corroborating the hypothesis, see Figure 1 above. The theoretical framework was developed using five recommended approaches: happiness, flexibility, clear employee engagement, training and development, and constant support. Each approach was mapped with one or many theories that relate to it, while contributing to employees' wellbeing while working from home. The framework offers a comprehensive and theoretically grounded approach to understanding the wellbeing of employees working from home. It emphasizes that wellbeing is a dynamic process shaped by organizational structures, relational support, and individual growth.

According to the developed theoretical framework, employers and employees should embrace a flexible hybrid work mode as a temporary arrangement during crises, as this supports both autonomy and human interactions, contributing to their happiness. Employees need to have clear roles and responsibilities to enhance their engagement. These roles and responsibilities should remain consistent and welldefined, even when employees are working from home. Training and development should not focus solely on technical and technological skills; it should also emphasize soft skills to facilitate a smooth transition between different work modes. Additionally, employers must provide ongoing support through regular meetings and briefing sessions to ensure employees feel connected and informed. This constant support fosters positive work relationships, a sense of belonging, safety, and affection, all of which contribute to a flourishing workplace.

Based on these insights, several recommendations can be made. Organizations should develop policies that promote a hybrid working model, as this approach fosters autonomy, flexibility, and employee satisfaction. However, these policies must be supported by internal procedures and processes to effectively manage employees working from home, ensuring sustained engagement and productivity. Additionally, employers should shift their training and development policies from a focus on technical skills to a more human-centered approach. This means prioritizing the needs of employees so that they can better cope with working remotely or in isolation.

Future research should focus on empirically validating the relationships between these dimensions and exploring how organizations can implement them in diverse and evolving work contexts. And finally, future research should consider quantitative, mixed-methods or longitudinal designs with larger, more diverse samples to validate and extend these qualitative insights, thereby enhancing the robustness and transferability of the proposed framework.

## Data Availability

The raw data supporting the conclusions of this article will be made available by the authors, without undue reservation.
